# Association of US Medical Marijuana Laws With Nonmedical Prescription Opioid Use and Prescription Opioid Use Disorder

**DOI:** 10.1001/jamanetworkopen.2019.7216

**Published:** 2019-07-17

**Authors:** Luis E. Segura, Christine M. Mauro, Natalie S. Levy, Nicole Khauli, Morgan M. Philbin, Pia M. Mauro, Silvia S. Martins

**Affiliations:** 1 Mailman School of Public Health, Department of Epidemiology, Columbia University, New York, New York; 2 Mailman School of Public Health, Department of Biostatistics, Columbia University, New York, New York; 3 Mailman School of Public Health, Department of Sociomedical Sciences, Columbia University, New York, New York

## Abstract

**Importance:**

Between 1997 and 2017, the United States saw increases in nonmedical prescription opioid use and its consequences, as well as changes in marijuana policies. Ecological-level research hypothesized that medical marijuana legalization may reduce prescription opioid use by allowing medical marijuana as an alternative.

**Objectives:**

To investigate the association of state-level medical marijuana law enactment with individual-level nonmedical prescription opioid use and prescription opioid use disorder among prescription opioid users and to determine whether these outcomes varied by age and racial/ethnic groups.

**Design, Setting, and Participants:**

This cross-sectional study used restricted data on 627 000 individuals aged 12 years and older from the 2004 to 2014 National Survey on Drug Use and Health, a population-based survey representative of the civilian population of the United States. Analyses were completed from March 2018 to May 2018.

**Exposures:**

Time-varying indicator of state-level medical marijuana law enactment (0 = never law enactment, 1 = before law enactment, and 2 = after law enactment).

**Main Outcomes and Measures:**

Past-year nonmedical prescription opioid use and prescription opioid use disorder among prescription opioid users. Odds ratios of nonmedical prescription opioid use and prescription opioid use disorder comparing the period before and after law enactment were presented overall, by age and racial/ethnic group, and adjusted for individual- and state-level confounders.

**Results:**

The study sample included 627 000 participants (51.51% female; 9.88% aged 12-17 years, 13.30% aged 18-25 years, 14.30% aged 26-34 years, 25.02% aged 35-49 years, and 37.50% aged ≥50 years; the racial/ethnic distribution was 66.97% non-Hispanic white, 11.83% non-Hispanic black, 14.47% Hispanic, and 6.73% other). Screening and interview response rates were 82% to 91% and 71% to 77%, respectively. Overall, there were small changes in nonmedical prescription opioid use prevalence after medical marijuana law enactment (4.32% to 4.86%; adjusted odds ratio, 1.13; 95% CI, 1.06-1.20). Prescription opioid use disorder prevalence among prescription opioid users decreased slightly after law enactment, but the change was not statistically significant (15.41% to 14.76%; adjusted odds ratio, 0.95; 95% CI, 0.81-1.11). Outcomes were similar when stratified by age and race/ethnicity.

**Conclusions and Relevance:**

This study found little evidence of an association between medical marijuana law enactment and nonmedical prescription opioid use or prescription opioid use disorder among prescription opioid users. Further research should disentangle the potential mechanisms through which medical marijuana laws may reduce opioid-related harm.

## Key Points

**Question:**

Is enactment of medical marijuana laws in the United States associated with changes in nonmedical prescription opioid use and prescription opioid use disorder among prescription opioid users overall and by age and racial/ethnic group?

**Findings:**

This cross-sectional study using individual-level restricted data from the 2004 to 2014 US National Survey on Drug Use and Health showed small increases in nonmedical prescription opioid use and no significant change in prescription opioid use disorder among users after medical marijuana law enactment. Similar patterns were observed across age and racial/ethnic groups.

**Meaning:**

Medical marijuana law enactment was not associated with a reduction in individual-level nonmedical prescription opioid use, contradicting the hypothesis that people would substitute marijuana for prescription opioids.

## Teaser

This cross-sectional study uses data from the US National Survey on Drug Use and Health to estimate changes in nonmedical prescription opioid use and prescription opioid use disorder in states that have legalized the use of medical marijuana.

## Introduction

Between 1999 and 2017, deaths from opioid overdose increased dramatically in the United States.^1^ An estimated 25 million US individuals aged 12 years and older initiated nonmedical use of prescription opioids (NMUPO) between 2002 and 2011.^2^ Furthermore, rates of opioid use disorder (OUD) have increased and in 2016, 2 million US individuals met the *Diagnostic and Statistical Manual of Mental Disorders* (Fourth Edition) definition of OUD.^3^ Research has partially attributed increases in OUD and opioid-related deaths to increases in prescription opioids dispensed for chronic, noncancer pain.^4^ Opioid prescribing increased from 1999 to 2010 and then decreased each year through 2015. However, opioid prescriptions remain approximately 3 times higher than in 1999.^5^ This spike in prescription opioids has contributed to 400 000 opioid overdose deaths in the United States between 1999 and 2017.^1^

Opioid prescriptions and NMUPO vary by racial/ethnic groups; non-Hispanic white individuals have higher prescription rates compared with other racial/ethnic groups.^5,6,7^ However, differences in opioid prescribing by race/ethnicity have narrowed in recent years.^8^ Trends in NMUPO also differ by age group and urbanicity.^9,10,11,12^ For instance, from 2002 to 2014, past-year NMUPO increased among those aged 12 to 17 years and 18 to 21 years and decreased among those aged 30 to 34 years.^12^

Parallel to the opioid crisis, and starting in 1996, marijuana-related policies have changed rapidly in the United States.^13,14,15^ Although specific regulations differ by state, medical marijuana laws (MML) permit the use of marijuana for medical purposes, primarily through the implementation of active dispensaries or allowances for home cultivation.^16^ As of November 2018, 33 US states had legalized medical marijuana, in addition to Washington, DC, Guam, and Puerto Rico.^15^

There is a growing literature on the medical use of marijuana as an alternative treatment for chronic pain.^17^ Existing research hypothesizes that the potential pain-alleviating benefit of marijuana, coupled with states’ legalization of medical marijuana, may offer an alternative to prescription opioids. Fewer prescriptions may, in turn, reduce opioid use, nonmedical use, and its consequences, eg, OUD and death.^18,19^ While multiple studies have investigated the effects of MMLs on marijuana use outcomes, research on MMLs’ effects on other substances—particularly prescription opioid use, misuse, and opioid-related harms—is scarce and of growing interest.

Some evidence suggests that MMLs may decrease some opioid-related outcomes.^18,19,20,21,22,23^ For example, ecological studies have found reductions in opioid overdose deaths after states enacted MMLs with dispensaries.^19,20^ Two studies^18,22^ using Medicaid data showed that state MMLs were associated with reductions in all prescriptions for conditions (eg, anxiety, depression, pain, psychosis, and seizures) for which medical marijuana could be an alternative. Similarly, a study^24^ using Medicaid data showed reductions in Schedule III opioid prescriptions (eg, codeine), number of prescriptions, dosage, and Medicaid spending on opioid prescriptions after MML implementation. An ecological study^19^ using aggregate data from the 2002 to 2012 National Survey on Drug Use and Health (NSDUH) found a slight inverse association between MML status and state-level aggregate prevalence of NMUPO and a decreasing trend in treatment admissions for OUD after states’ enactment of MMLs with dispensaries. However, these studies used administrative, state-level data and could not determine the association of MMLs with individual NMUPO because results from ecological studies do not necessarily mirror individual-level behavior.

To our knowledge, only 1 study has examined the association between MML status and NMUPO. This study used individual-level data from 1991 to 2015 and showed a reduction in NMUPO among eighth-grade students after enactment of state MMLs.^23^ Similar studies exploring the association of the changing policy environment with individual NMUPO including a wider range of ages have not been conducted to date.

This study used a national and state representative sample from 2004 to 2014 to investigate the association between living in a state with MML enactment and individual-level NMUPO and prescription opioid use disorder (POUD) among nonmedical prescription opioid (PO) users. Moreover, we estimated these outcomes by age and racial/ethnic groups. We hypothesized that state legalization of medical marijuana would be associated with decreases in both NMUPO and POUD among nonmedical PO users. In addition, we expected the associations of MML with NMUPO and POUD among nonmedical PO users to be more pronounced among non-Hispanic white individuals and among individuals aged 26 to 34 years, as these groups experience the highest prevalences of NMUPO and POUD.

## Methods

### Study Sample

We used data from the 2004 to 2014 NSDUH restricted-use data portal. The NSDUH is a complex multistage probability survey conducted annually among the US civilian noninstitutionalized population aged 12 years and older (approximately 70 000 individuals yearly). This survey produced national and state representative estimates of substance use behaviors. The NSDUH survey design included 50 states and the District of Columbia with an independent multistage area probability sample for each state and the District of Columbia. Age groups 12 to 17 years and 18 to 25 years were oversampled to create equal-sized samples among individuals aged 12 to 17 years, 18 to 25 years, and 26 years and older. To guarantee privacy and honest reporting of illegal drug use and other sensitive behaviors, interviews were conducted by trained interviewers using computer-assisted personal interviewing and audio computer-assisted self-interviewing. Survey weights generate unbiased estimates representing the target US population each year and additionally adjust for selection probabilities at each stage and for nonresponse, coverage, or extreme weights. Screening and interview response rates varied from 82% to 91% and 71% to 77%, respectively.^25^ This study was considered non–human subjects research by the Columbia University institutional review board because data were deidentified. Reporting of this study follows the Strengthening the Reporting of Observational Studies in Epidemiology (STROBE) reporting guideline for cross-sectional studies.^26^

### Measures and Procedures

The primary exposure was state-level MML enactment status. We used 3 different operationalizations of MML: 1 for visually assessing trends over time, and the other 2 as the main exposures in our models. For trends, states were categorized as (1) never passing MML (never MML pass), (2) enacting MML before 2004 (early MML pass), and (3) enacting MML after 2004 (late MML pass). In our first set of models, we coded MML using a time-varying 3-level predictor (never MML enactment, before MML enactment, and after MML enactment). For example, living in the state of Delaware before 2011 was coded as “before MML enactment” and living in the state of Delaware in 2011 and after were coded as “after MML enactment.” The Figure displays states’ MML categorization over time. In a second set of models (eTable 1 and eTable 2 in the Supplement), we further divided the category “after MML enactment” into “after MML enactment without dispensaries” and “after MML enactment with dispensaries.”

**Figure.**
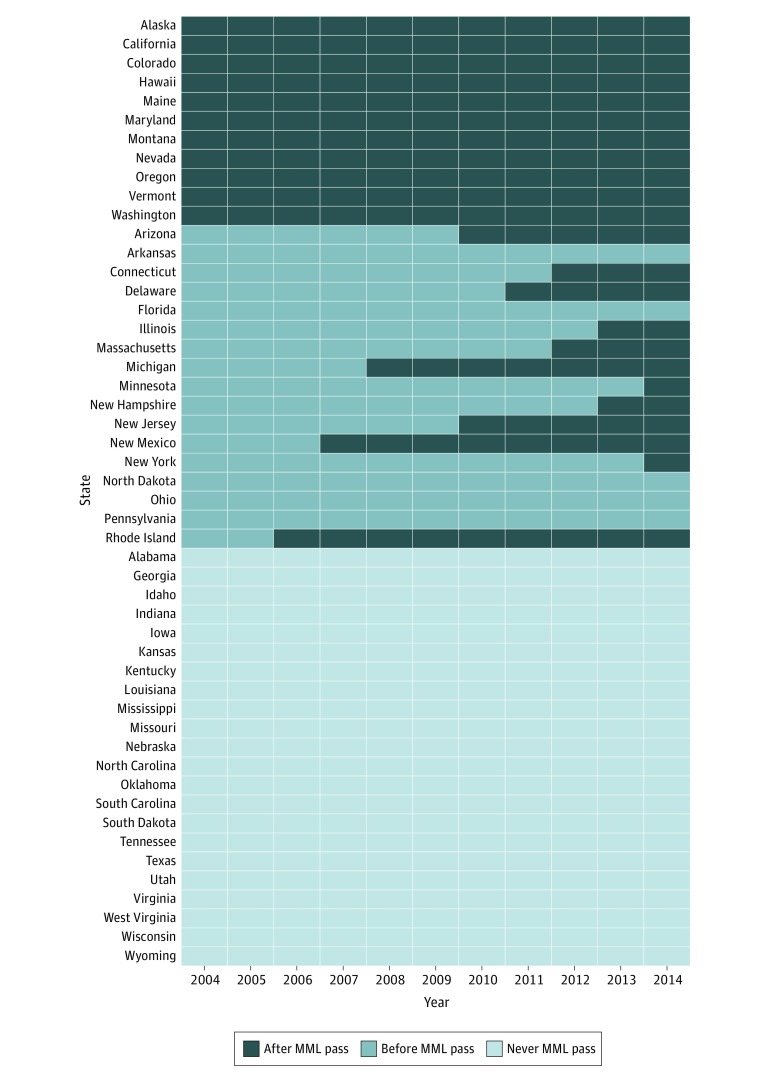
Enactment Status of Medical Marijuana Laws (MMLs) for Each US State

Our 2 outcomes of interest were past-year NMUPO and past-year POUD among nonmedical PO users. The NSDUH data from 2004 to 2014 only included questions about NMUPO. Information on medical use of prescription opioids was not available in the NSDUH in the years analyzed for this article; such questions were first included in the NSDUH 2015, so estimates derived from those questions were not comparable to earlier years. In NSDUH 2004 to 2014, all participants were first provided with a flashcard with prescription opioid names and pictures—these excluded nonopioid pain medications—and were asked to indicate if they had used any of these prescription opioids. Participants who endorsed any prescription opioid use were then asked “How long has it been since you last used any prescription pain reliever that was not prescribed for you or that you took only for the experience or feeling it caused?” The NSDUH provided an imputed revised binary indicator of past-year NMUPO (no or yes use) derived from these 2 questions. Similarly, the NSDUH includes structured questions based on the *Diagnostic and Statistical Manual of Mental Disorders* (Fourth Edition) criteria for substance disorders.^27^ Opioid use disorder secondary to NMUPO is defined by whether the respondent endorsed 1 of 4 abuse questions and/or 3 of the dependence items in the past year. This indicator of POUD (0 = no and 1 = yes) has good agreement with clinical judgment.^28^

We included individual- and state-level predictors as confounding control variables. Individual-level predictors included a binary indicator of sex (male or female); a 5-category indicator of age (12-17, 18-25, 26-34, 35-49, and ≥50 years); racial/ethnic group (non-Hispanic white, non-Hispanic black, Hispanic, and non-Hispanic other); survey year (2004-2014); any health insurance coverage (yes or no); family income (<$20 000; $20 000-$49 999; $50 000-$74 999; or ≥$75 000), and population density based on location of individual’s household according to core-based statistical area (CBSA) (CBSA with >1 million persons, CBSA with <1 million persons, or not in a CBSA); state of residence indicator; and binary indicator of living in a state with currently enacted prescription drug monitoring program laws.

State-level predictors included proportion of the state’s population that was white, male, aged 10 to 24 years, and aged 25 years or older with at least a high school education. We also included state-level indicators of unemployment rate and median household income.

### Statistical Analysis

To describe the prevalence of NMUPO and POUD among nonmedical PO users over time, we calculated weighted prevalences of NMUPO and POUD among nonmedical PO users in each survey year from 2004 to 2014 by averaging NSDUH-weighted state-level prevalence estimates across each of the 3 MML state indicators: never MML pass, late MML pass, and early MML pass. The variances of the prevalence estimates were calculated using the Taylor linearization method, which incorporated survey weights to account for the NSDUH complex survey design.^29^ Prevalence estimates were also stratified by racial/ethnic group and age group.

The main analyses used multilevel logistic regression with state-level indicators as random effects to test the association between (1) MMLs and NMUPO and (2) MMLs and POUD among nonmedical PO users. Our main contrast of interest was the comparison of the before and after MML passage period on NMUPO and POUD among nonmedical PO users. As such, we computed adjusted odds ratios (aORs) and 95% confidence intervals comparing the categories after MML passage vs before MML passage by separately regressing NMUPO (model 1) and POUD (model 2) on the time-varying indicator of MML (never MML passage, before MML passage, and after MML passage) and adjusting for individual- and state-level predictors. The models used data from all 50 states, including states that did not pass MML, to control for historical time trends in NMUPO and POUD among nonmedical PO users across the period of 2004 to 2014. This allows us to estimate the change before and after MML passage above and beyond what would have been expected had no MML been passed, similar to a difference-in-differences approach. In all models, time was included as a continuous variable using a piecewise spline function of year with a knot in 2008. The knot at 2008 was chosen based on previous research on the association of MML with marijuana use using the same year as a knot^30^ and on our own exploratory analysis showing a decrease in NMUPO and increase in POUD among nonmedical PO users after this period (eFigure 1 and eFigure 2 in the Supplement). To obtain estimates by age and racial/ethnic group, we separately added interaction terms between the exposure indicator of MML and age group, and MML and racial/ethnic group, to the original NMUPO (models 3 and 4) and POUD among nonmedical PO users (models 5 and 6) models. Additionally, we ran these same models using the more expansive MML exposure definition (never MML passage, before MML passage, after MML passage with dispensaries, and after MML passage without dispensaries) to estimate aORs comparing the category after the passage of MML with categories before passage of MML both with and without dispensaries. Survey weights were not included in our models because we included all individual-level indicators related to the sampling design.^31^

For nonnull results with confidence intervals that did not cross 1, we conducted a sensitivity analysis to quantify the minimum strength of association that unmeasured and unobserved confounding—the E-value—would need to have with both the exposure and the outcome, above and beyond adjustment for measured and observed confounders, to reduce the adjusted point estimates (aORs) to the null (eTable 3 in the Supplement).^32^ We also estimated E-values for the lower limit of the confidence intervals to test how robust our findings were to the presence of unmeasured confounding that would bias the 95% confidence interval to include the null.^32^ The E-value is measured in the scale of the observed exposure-outcome association. To illustrate the usefulness of the E-value we provide the following hypothetical example. Assume that exposure and outcome are associated by an OR of 1.30, and the computed E-value for such association is 1.20. Then the E-value interpretation is that the observed OR of 1.30 could be explained away by unmeasured confounding that was associated with both exposure and outcome by an OR of 1.20 each above and beyond adjustment for measured confounders. This weak and plausible magnitude of unmeasured confounding association (OR = 1.20) could explain away the observed association.^32^ On the other hand, an unmeasured confounding association by an OR of 3.0 (E-value) would provide evidence of a more robust estimate to unmeasured confounding. When assessing for trends, *P* values for trend were obtained using 2-tailed Wald tests.

All analyses were conducted using a Linux-based SAS statistical software version 9.4 grid system (SAS Institute Inc) available in the Substance Abuse and Mental Health Services Administration data portal. Sensitivity analyses and figures were conducted in R statistical software version 3.5.2 (R Project for Statistical Computing) using the EValue^33^ and ggplot2^34^ packages.

## Results

Screening and interview response rates were 82% to 91% and 71% to 77%, respectively. The final analytical 2004 to 2014 NSDUH sample comprised 627 000 individuals (48.49% male and 51.51% female). The racial/ethnic distribution was 66.97% non-Hispanic white, 11.83% non-Hispanic black, 14.47% Hispanic, and 6.73% other. The age group distribution was 9.88% 12 to 17 years, 13.30% 18 to 25 years, 14.30% 26 to 34 years, 25.02% 35 to 49 years, and 37.50% 50 years or older. Descriptive results showing trends over time of NMUPO and POUD among nonmedical PO users by state are included in eFigure 1 to eFigure 6 in the Supplement. The prevalence of NMUPO decreased between 2004 and 2014 for never MML states (4.66% to 3.94%), early MML states (4.94% to 4.24%), and late MML states (4.57% to 3.68%). Decreasing trends in NMUPO also occurred among those aged 12 to 17 years and 18 to 25 years and among non-Hispanic white individuals. There was a slight increase in NUMPO among those older than 50 years and among non-Hispanic black individuals. Among nonmedical PO users, POUD increased slightly during the study period across all states regardless of MML enactment—from 15.36% to 17.76% in never MML states, from 11.41% to 13.99% in early MML states, and from 12.03% to 17.94% in late MML states. There were no discernible POUD trends among nonmedical PO users by age group; there were slight increases among non-Hispanic white individuals in states with early and late MML enactment.

Table 1 compares the prevalence of NMUPO before and after state enactment of MML between 2004 and 2014. Overall, the prevalence of NMUPO increased from 4.32% to 4.86% following state MML enactment (aOR, 1.13; 95% CI, 1.06-1.20). The change in the prevalence of NMUPO from after vs before the passage of MML ranged between 0.14% to 0.54% across age groups. The prevalence of NMUPO also increased slightly for all racial/ethnic groups except non-Hispanic other. Similar results were seen when comparing the associations of MML enactment in states with and without dispensaries (eTable 1 in the Supplement).

**Table 1.** Comparison of Past-Year Nonmedical Prescription Opioid Use Between States Before MML Passage and After MML Passage

Characteristic	Nonmedical Prescription Opioid Use, %		Adjusted Odds Ratio (95% CI)^a^
	Before MML	After MML	
All individuals	4.32	4.86	1.13 (1.06-1.20)
Age, y			
12-17	5.81	6.13	1.06 (0.99-1.13)
18-25	9.40	9.54	1.02 (0.96-1.08)
26-34	6.04	6.32	1.05 (0.97-1.14)
35-49	3.51	4.01	1.15 (1.05-1.25)
≥50	1.24	1.75	1.42 (1.23-1.64)
Race/ethnicity			
Non-Hispanic white	6.20	6.53	1.06 (1.00-1.12)
Non-Hispanic black	3.49	4.38	1.26 (1.14-1.39)
Hispanic	3.53	4.22	1.21 (1.11-1.31)
Non-Hispanic other	4.55	4.59	1.01 (0.92-1.11)

Abbreviation: MML, medical marijuana law.

^a^ Models were adjusted for MML status (never, before, after); individual-level predictors, including racial/ethnic group, age group, time as a continuous variable using a piecewise spline function of year with a knot in 2008, sex, any health insurance coverage, family income, population density based on location of individual’s household, state of residence indicator, and an indicator of living in a state with currently enacted Prescription Drug Monitoring Program laws; and state-level predictors, including the proportion of individuals in the state who were white, male, ages 10 to 24 years, and aged 25 years with at least a high school education, as well as unemployment and state’s median household income.

Table 2 shows the after vs before effect of state MML enactment on POUD among nonmedical PO users. Overall, the prevalence of POUD among nonmedical PO users after state MML enactment slightly decreased from 15.41% to 14.76%, but the change was not statistically significant (aOR, 0.95; 95% CI, 0.81-1.11). The prevalence of POUD among nonmedical PO users after MML enactment decreased for those aged 18 to 25 years and 26 to 34 years and increased slightly among those aged 35 to 49 years and those 50 years or older; however, the aORs showed no statistically significant associations. When exploring the associations by race/ethnicity, we observed a decrease in the prevalence of POUD after vs before MML enactment by racial/ethnic group that ranged from −1.47% to 0.24%. The prevalence of POUD among nonmedical PO users after state MML enactment decreased somewhat among non-Hispanic white individuals (aOR, 0.98; 95% CI, 0.86-1.13), non-Hispanic black individuals (aOR, 0.87; 95% CI, 0.65-1.16), and non-Hispanic individuals of other race (aOR, 0.94; 95% CI, 0.73-1.20) but increased among Hispanic individuals (aOR, 1.02; 95% CI, 0.83-1.26), but the changes were not statistically significant. Findings were similar when states with MML enactment were further classified by dispensary status (eTable 2 in the Supplement).

**Table 2.** Comparison of Past-Year Prescription Opioid Use Disorder Among Nonmedical Prescription Opioid Users Between States Before MML Passage and After MML Passage

Characteristic	Prescription Opioid Use Disorder, %		Adjusted Odds Ratio (95% CI)^a^
	Before MML	After MML	
All users	15.41	14.76	0.95 (0.81-1.11)
Age, y			
12-17	16.08	14.93	0.92 (0.78-1.08)
18-25	14.71	14.46	0.98 (0.85-1.13)
26-34	17.22	14.64	0.82 (0.66-1.03)
35-49	15.09	15.22	0.99 (0.78-1.26)
≥50	14.10	14.56	1.03 (0.68-1.54)
Race/ethnicity			
Non-Hispanic white	17.26	17.02	0.98 (0.86-1.13)
Non-Hispanic black	12.52	11.05	0.87 (0.65-1.16)
Hispanic	14.64	14.88	1.02 (0.83-1.26)
Non-Hispanic other	17.70	16.79	0.94 (0.73-1.20)

Abbreviation: MML, medical marijuana law.

^a^ Models were adjusted for MML status (never, before, after); individual-level predictors, including racial/ethnic group, age group, time as a continuous variable using a piecewise spline function of year with a knot in 2008, sex, any health insurance coverage, family income, population density based on location of individual’s household, state of residence indicator, and an indicator of living in a state with currently enacted Prescription Drug Monitoring Program laws; and state-level predictors, including the proportion of individuals in the state who were white, male, ages 10 to 24 years, and aged 25 years with at least a high school education, as well as unemployment and state’s median household income.

Sensitivity analysis to estimate the impact of unmeasured and unobserved confounding on nonnull adjusted associations (Table 1 and Table 2; eTable 1 and eTable 2 in the Supplement) and their corresponding lower level confidence interval can be seen in eTable 3 in the Supplement. The minimum strength of association that an unmeasured and unobserved confounder would need to have with both the exposure—MML—and outcome (NMUPO or POUD) to reduce these associations and their respective lower level confidence interval to the null was small—the range of E-values for nonnull ORs was 1.40 to 2.43 and the range of E-values for confidence intervals crossing 1 was 1.11 to 1.92.

## Discussion

In this study, we investigated the associations between MML enactment (with and without dispensaries) and changes in NMUPO and POUD among nonmedical PO users, and whether these associations differed by age and racial/ethnic group. When comparing the overall effect of after vs before MML enactment, we found small increases in NMUPO and slight decreases or no change in POUD among nonmedical PO users—even for states with MML that allowed dispensaries. This association was similar across all age and racial/ethnic groups. To the extent that residual confounding and chance may explain these small estimates, the enactment of MML did not seem to have a clinically meaningful association with NMUPO and POUD among nonmedical PO users, even when examining whether a state MML allowed dispensaries. Thus, living in a state with MML on NMUPO and POUD among nonmedical PO users is compatible with a null outcome.

There are a number of potential reasons for the discrepancy between our results and previous studies that found decreases in other opioid measures after MML. Previous research includes primarily ecological-level studies that used indirect and aggregate measures of state-level NMUPO—such as state-level prescription rates of opioid medications and dosages—and its consequences (ie, treatment admissions for opioid addiction and opioid-related deaths).^18,19,20,21,22,23,24^ These studies have demonstrated an inverse association between state enactment of MML and state prevalence of NMUPO and POUD.^18,19,20,21,22^ These studies have hypothesized that reductions in state prevalence of NMUPO and POUD following MML enactment may be due to health care professionals in MML states reducing opioid prescribing. This is thought to be explained by health care professionals recommending the use of medical marijuana instead of opioid medications, which might decrease individual NMUPO and its consequences—such as POUD.^18,20,21,22^ However, we found no evidence that MML enactment—even with dispensaries—was associated with a decrease in individual NMUPO. On the contrary, we found evidence of small, although mostly not statistically significant, increases in individual reports of NMUPO both overall and by age and racial/ethnic group after MML enactment. In addition, we found no evidence, indicated by null estimates, to suggest that MML enactment may decrease individual POUD. Our findings suggest that previous findings of a reduction in the prevalence of opioid-related harms following MML enactment^18,19,20,21,22,23,24^ might not occur through the reduction of individual misuse of prescription opioids. Moreover, as previous studies note, the associations between MML exposures and NMUPO obtained at the ecological level may not reflect individual-level behaviors.^35^ Research should explore the specific mechanisms that may contribute to the potential preventive effect of MML on NMUPO and its consequences.

### Limitations

We note some limitations to the study. Measures of NMUPO and POUD—our main outcomes—were self-reported, so there is potential measurement error. The NSDUH was designed to reduce the underreporting of substance use and related measures by using computer-based techniques.^25,36^ We could not explore more recent years in the NSDUH (2015-2017) owing to methodological changes in the NSDUH’s opioid-related module questions after 2014. Despite the fact that the NSDUH is representative of the US civilian population, this survey excludes some important populations, including homeless and incarcerated individuals and active military personnel.^36^ While we incorporated whether MMLs allow dispensaries, we did not explore associations between other potential state-level differences in MMLs and NMUPO and POUD among nonmedical PO users. Additionally, our analysis did not control for the effect of enacted recreational marijuana legalization. However, we believe the impact of this potential unmeasured confounder was unlikely to bias our estimates as only 2 states (Washington and Colorado) approved legal use of marijuana in 2012,^37^ and enacted the law in 2014.^38^ Others like Alaska, Oregon, and Washington, DC, followed in 2014—the end of our study period.^39^ Nonetheless, this study has multiple strengths. The NSDUH is a nationally representative survey of the US civilian population. It provides high-quality data about substance use and mental health. Owing to the oversampling design, age groups are well represented; to our knowledge, the NSDUH is the only survey on drug use that includes participants as young as 12 years. Our estimates were derived from the restricted-use individual-level 2004 to 2014 NSDUH, which were adjusted for potential confounders at both the individual and state levels.

## Conclusions

Study findings do not support the hypothesis that living in a state with MMLs reduces individual NMUPO and POUD. We found small increases in NMUPO and small decreases in POUD among nonmedical PO users after MML enactment, or, at best, no association, even among MML states with dispensaries. The contradiction between our results and previous evidence may be explained by previous use of indirect or aggregate NMUPO measures, with existing studies using ecological-level data. Our results suggest that if MMLs do have any effect on opioid-related outcomes, it likely does not occur through reducing individual-level NMUPO. Further research should disentangle the potential mechanisms through which living in a state that has enacted MMLs may be associated with reduced opioid-related harms. If our results are replicated in future studies, this could suggest that medical marijuana policies may be insufficient to reduce individual-level opioid outcomes. Instead, it would suggest that opioid-specific approaches and policy interventions (ie, prescription drug monitoring programs, prescribing practices laws, pain clinic laws) are needed.

## Notes

Supplement. **eFigure 1.** Prevalence of Nonmedical Prescription Opioid Use by States That Passed MMLs Before 2004 (Early MML Pass), After 2004 (Late MML Pass), or Never Passed MML **eFigure 2.** Prevalence of Prescription Opioid Use Disorder Among Prescription Opioid Users by States That Passed MMLs Before 2004 (Early MML Pass), After 2004 (Late MML Pass), or Never Passed MML **eFigure 3.** Prevalence of Nonmedical Prescription Opioid Use by Age Group Among States That Passed MML Before 2004 (Early MML Pass), After 2004 (Late MML Pass) or Never Passed MML **eFigure 4.** Prevalence of Nonmedical Prescription Opioid Use by Race/Ethnic Group in States That Passed MML Before 2004 (Early MML Pass), After 2004 (Late MML Pass) or Never Passed MML **eFigure 5.** Prevalence of Prescription Opioid Use Disorder Among Prescription Opioid Users by Age Group Among States That Passed MML Before 2004 (Early MML Pass), After 2004 (Late MML Pass) or Never Passed MML **eFigure 6.** Prevalence of Prescription Opioid Use Disorder Among Prescription Opioid Users by Race/Ethnic Group in States That Passed MML Before 2004 (Early MML Pass), After 2004 (Late MML Pass) or Never Passed MML **eTable 1.** Comparison of Past-Year Nonmedical Prescription Opioid Use Comparing People in States Before MML Passage and After MML Passage Without Dispensaries, and Between States Before MML Passage and After MML Passage With Dispensaries **eTable 2.** Comparison of Past-Year Prescription Opioid Use Disorder Among Nonmedical Prescription Opioid Users Between States Before MML Passage and After MML Passage Without Dispensaries, and Between States Before MML Passage and After MML Passage With Dispensaries **eTable 3.** Sensitivity Analyses to Quantify the Minimum Amount of Unmeasured Confounding Required to Nullify the Associations Between NMUPO and MML Passage, and POUD Among PO Users and MML Passage
