# Supplementary material for: Association of US Medical Marijuana Laws With Nonmedical Prescription Opioid Use and Prescription Opioid Use Disorder
Source: JAMA Netw Open. 2019 Jul 17;2(7):e197216. doi: 10.1001/jamanetworkopen.2019.7216 (PMC6647549; doi:10.1001/jamanetworkopen.2019.7216)
Supplement: Supplement. — eFigure 1. Prevalence of Nonmedical Prescription Opioid Use by States That Passed MMLs Before 2004 (Early MML Pass), After 2004 (Late MML Pass), or Never Passed MML eFigure 2. Prevalence of Prescription Opioid Use Disorder Among Prescription Opioid Users by States That Passed MMLs Before 2004 (Early MML Pass), After 2004 (Late MML Pass), or Never Passed MML eFigure 3. Prevalence of Nonmedical Prescription Opioid Use by Age Group Among States That Passed MML Before 2004 (Early MML Pass), After 2004 (Late MML Pass) or Never Passed MML eFigure 4. Prevalence of Nonmedical Prescription Opioid Use by Race/Ethnic Group in States That Passed MML Before 2004 (Early MML Pass), After 2004 (Late MML Pass) or Never Passed MML eFigure 5. Prevalence of Prescription Opioid Use Disorder Among Prescription Opioid Users by Age Group Among States That Passed MML Before 2004 (Early MML Pass), After 2004 (Late MML Pass) or Never Passed MML eFigure 6. Prevalence of Prescription Opioid Use Disorder Among Prescription Opioid Users by Race/Ethnic Group in States That Passed MML Before 2004 (Early MML Pass), After 2004 (Late MML Pass) or Never Passed MML eTable 1. Comparison of Past-Year Nonmedical Prescription Opioid Use Comparing People in States Before MML Passage and After MML Passage Without Dispensaries, and Between States Before MML Passage and After MML Passage With Dispensaries eTable 2. Comparison of Past-Year Prescription Opioid Use Disorder Among Nonmedical Prescription Opioid Users Between States Before MML Passage and After MML Passage Without Dispensaries, and Between States Before MML Passage and After MML Passage With Dispensaries eTable 3. Sensitivity Analyses to Quantify the Minimum Amount of Unmeasured Confounding Required to Nullify the Associations Between NMUPO and MML Passage, and POUD Among PO Users and MML Passage [file jamanetwopen-2-e197216-s001.pdf]

Supplementary Online Content

Segura LE, Mauro CM, Levy NS, et al. Association of US medical marijuana laws with nonmedical prescription opioid use and prescription opioid use disorder. *JAMA Netw Open*. 2019;2(7):e197216. doi:10.1001/jamanetworkopen.2019.7216

eFigure 1. Prevalence of Nonmedical Prescription Opioid Use by States That Passed MMLs Before 2004 (Early MML Pass), After 2004 (Late MML Pass), or Never Passed MML
eFigure 2. Prevalence of Prescription Opioid Use Disorder Among Prescription Opioid Users by States That Passed MMLs Before 2004 (Early MML Pass), After 2004 (Late MML Pass), or Never Passed MML
eFigure 3. Prevalence of Nonmedical Prescription Opioid Use by Age Group Among States That Passed MML Before 2004 (Early MML Pass), After 2004 (Late MML Pass) or Never Passed MML
eFigure 4. Prevalence of Nonmedical Prescription Opioid Use by Race/Ethnic Group in States That Passed MML Before 2004 (Early MML Pass), After 2004 (Late MML Pass) or Never Passed MML
eFigure 5. Prevalence of Prescription Opioid Use Disorder Among Prescription Opioid Users by Age Group Among States That Passed MML Before 2004 (Early MML Pass), After 2004 (Late MML Pass) or Never Passed MML
eFigure 6. Prevalence of Prescription Opioid Use Disorder Among Prescription Opioid Users by Race/Ethnic Group in States That Passed MML Before 2004 (Early MML Pass), After 2004 (Late MML Pass) or Never Passed MML
eTable 1. Comparison of Past-Year Nonmedical Prescription Opioid Use Comparing People in States Before MML Passage and After MML Passage Without Dispensaries, and Between States Before MML Passage and After MML Passage With Dispensaries
eTable 2. Comparison of Past-Year Prescription Opioid Use Disorder Among Nonmedical Prescription Opioid Users Between States Before MML Passage and After MML Passage Without Dispensaries, and Between States Before MML Passage and After MML Passage With Dispensaries
eTable 3. Sensitivity Analyses to Quantify the Minimum Amount of Unmeasured Confounding Required to Nullify the Associations Between NMUPO and MML Passage, and POUD Among PO Users and MML Passage

This supplementary material has been provided by the authors to give readers additional information about their work.

© 2019 Segura LE et al. *JAMA Network Open.*

eFigure 1. Prevalence of nonmedical prescription opioid use by states that passed MMLs before 2004 (Early MML Pass), after 2004 (Late MML Pass), or never passed MML.

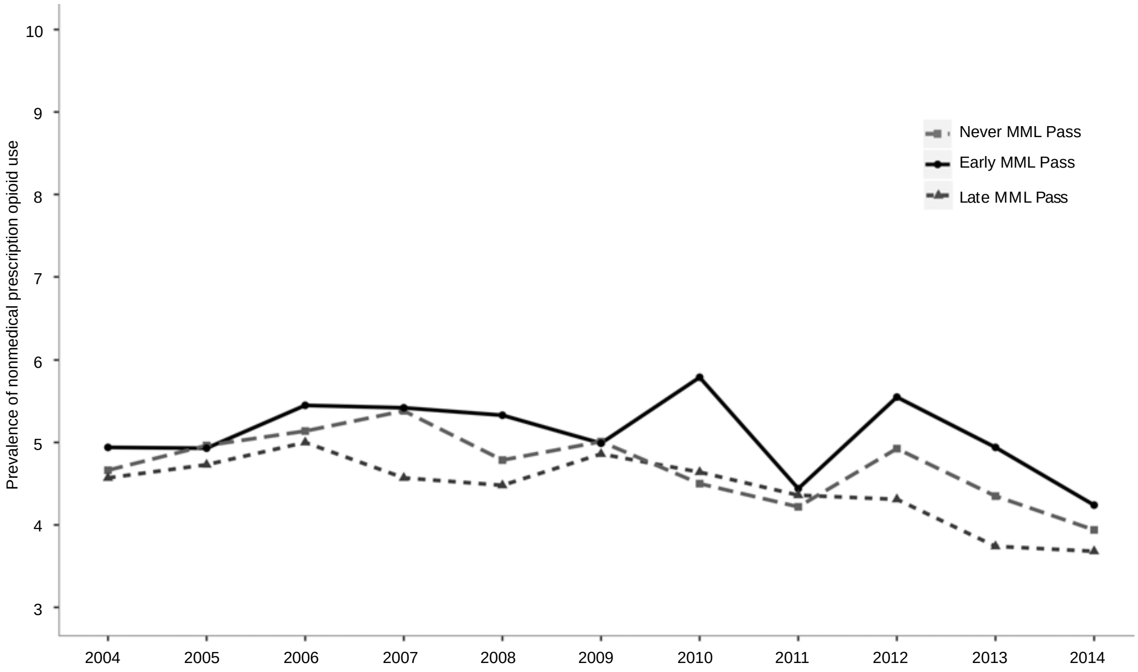

Never MML Pass p-for-trend < 0.05

Early MML Pass p-for-trend > 0.05

Late MML Pass p-for-trend < 0.05

MML: Medical Marijuana Laws

© 2019 Segura LE et al. *JAMA Network Open.*

eFigure 2. Prevalence of prescription opioid use disorder among prescription opioid users by states that passed MMLs before 2004 (Early MML Pass), after 2004 (Late MML Pass), or never passed MML.

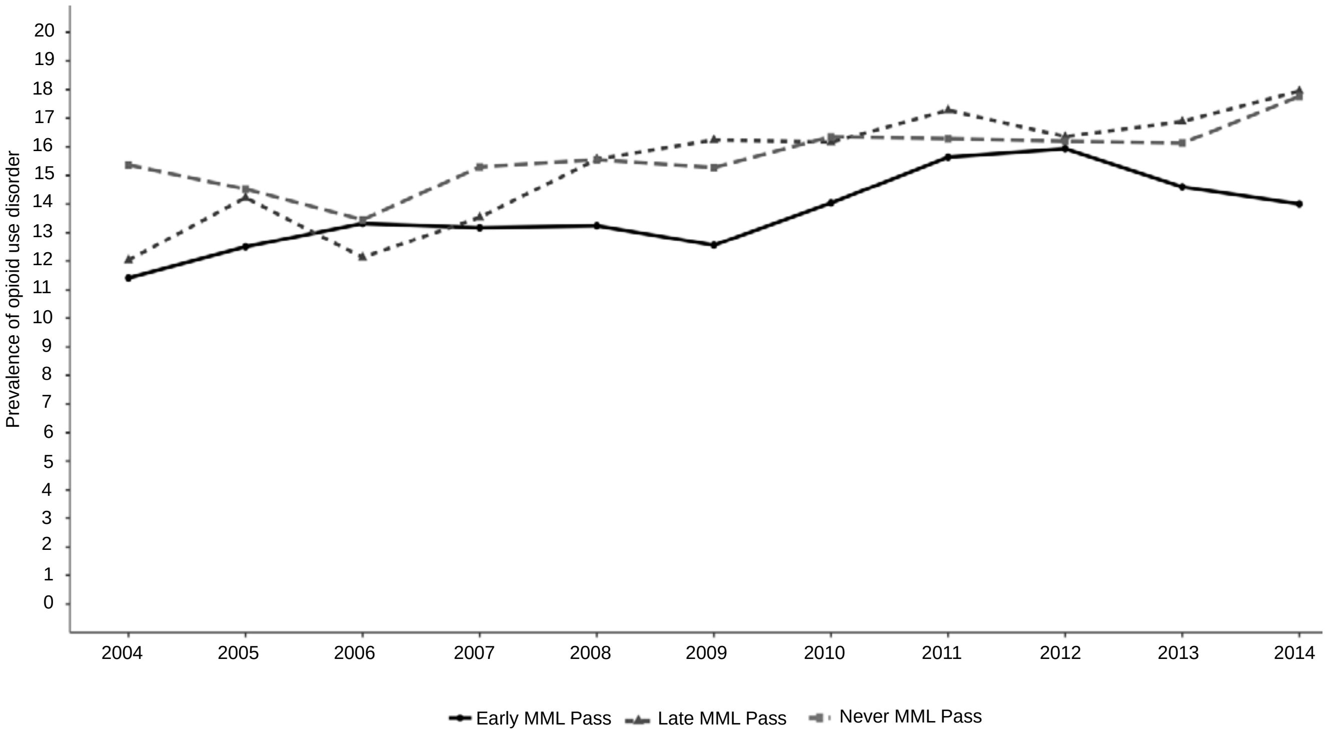

Never MML Pass p-for-trend > 0.05

Early MML Pass p-for-trend > 0.05

Late MML Pass p-for-trend > 0.05

MML: Medical Marijuana Laws

© 2019 Segura LE et al. *JAMA Network Open.*

eFigure 3. Prevalence of nonmedical prescription opioid use by age group among states passed MML before 2004 (Early MML Pass), after 2004 (Late MML Pass) or never passed MML.

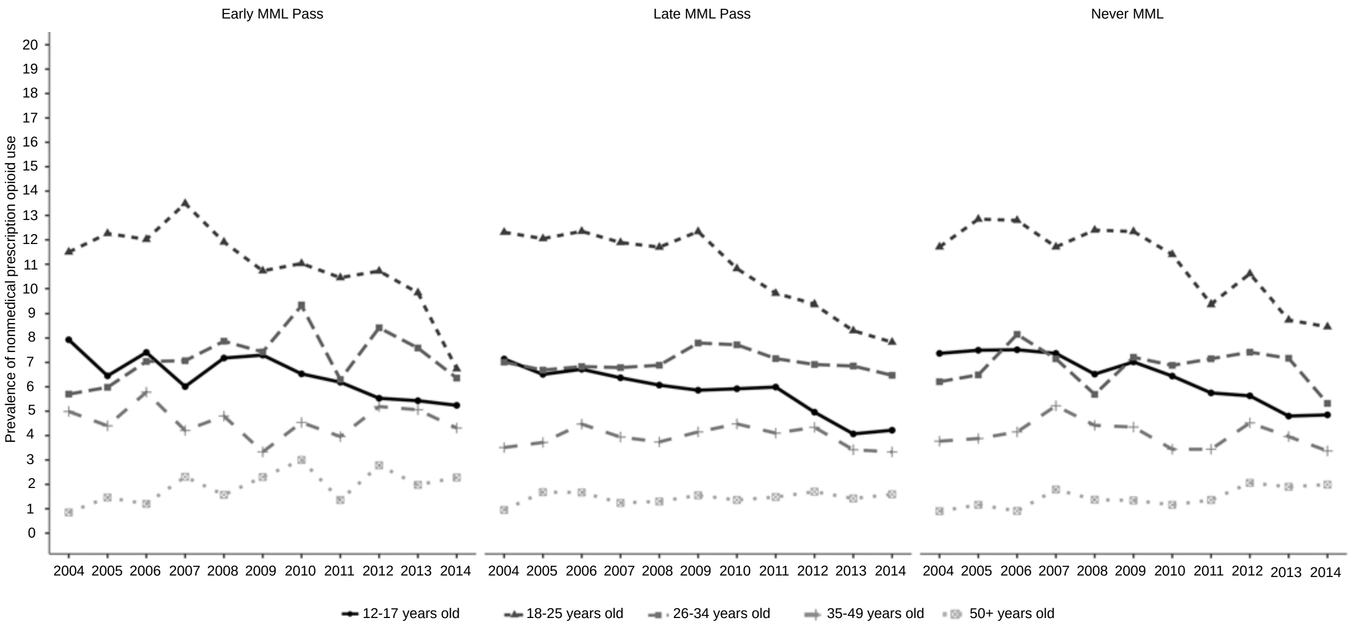

p-for-trend < 0.05 among 12-17 years old in states that never passed an MML, states that passed MML early, and states that passed MML late.

p-for-trend < 0.05 among 18-25 years old in states that never passed an MML, states that passed MML early, and states that passed MML late.

p-for-trend > 0.05 among 26-34 years old in states that never passed an MML, states that passed MML early, and states that passed MML late.

p-for-trend > 0.05 among 35-49 years old in states that never passed an MML, states that passed MML early, and states that passed MML late.

p-for-trend < 0.05 among 50+ years old in states that never passed an MML and in states that passed MML early, and p-for-trend > 0.05 in states that passed MML late.

MML: Medical Marijuana Laws

© 2019 Segura LE et al. *JAMA Network Open.*

eFigure 4. Prevalence of nonmedical prescription opioid use by race/ethnic group in states that passed MML before 2004 (Early MML Pass), after 2004 (Late MML Pass) or never passed MML.

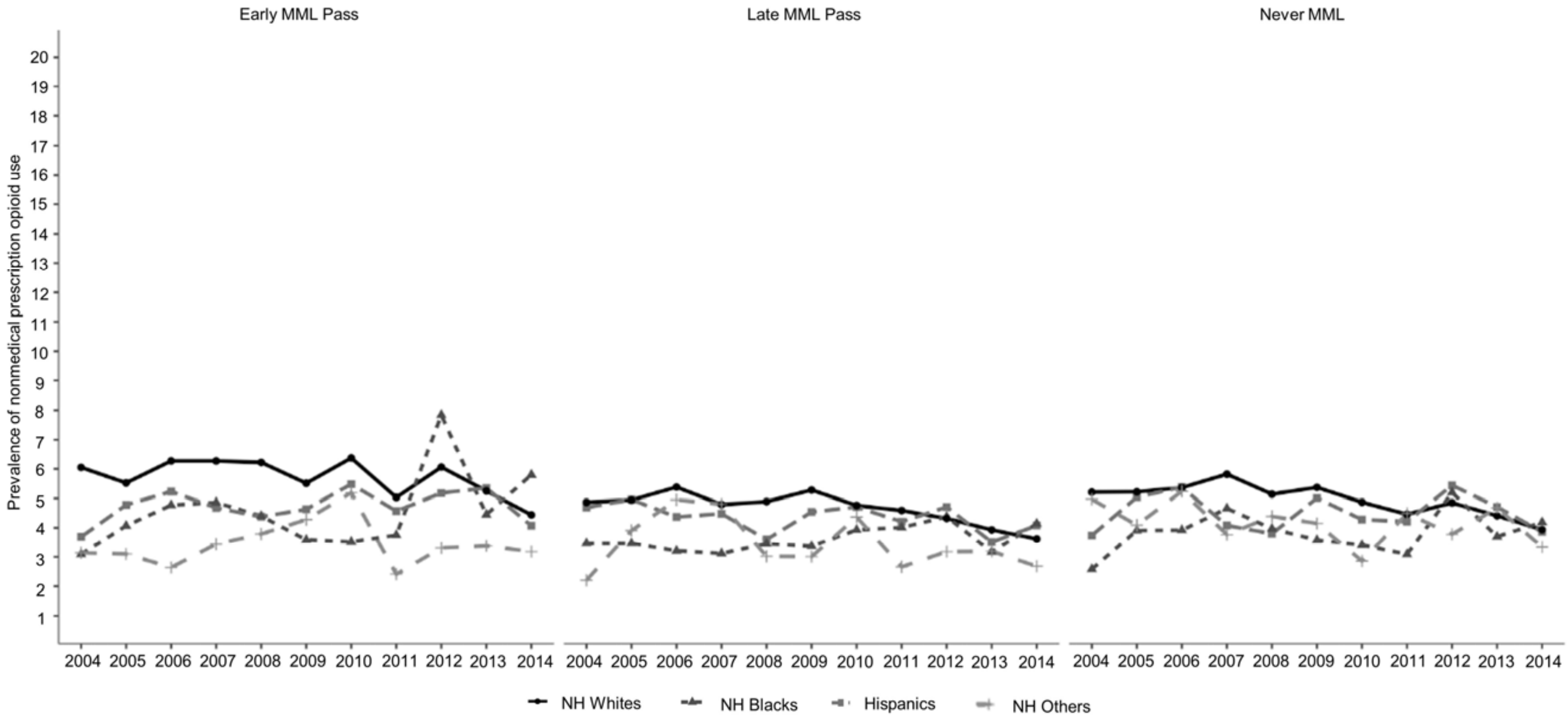

P-for-trend < 0.05 among NH Whites in states that never passed MML and states that passed MML late, and p-for-trend > 0.05 in states that passed MML early.

P-for-trend > 0.05 among NH Blacks in states that never passed MML, states that passed MML early, and states that passed MML late.

P-for-trend > 0.05 among Hispanics in states that never passed MML, states that passed MML early, and states that passed MML late.

P-for-trend > 0.05 among NH Others in states that never passed MML, states that passed MML early, and states that passed MML late.

MML: Medical Marijuana Laws

© 2019 Segura LE et al. *JAMA Network Open.*

eFigure 5. Prevalence of prescription opioid use disorder among prescription opioid users by age group among states passed MML before 2004 (Early MML Pass), after 2004 (Late MML Pass) or never passed MML.

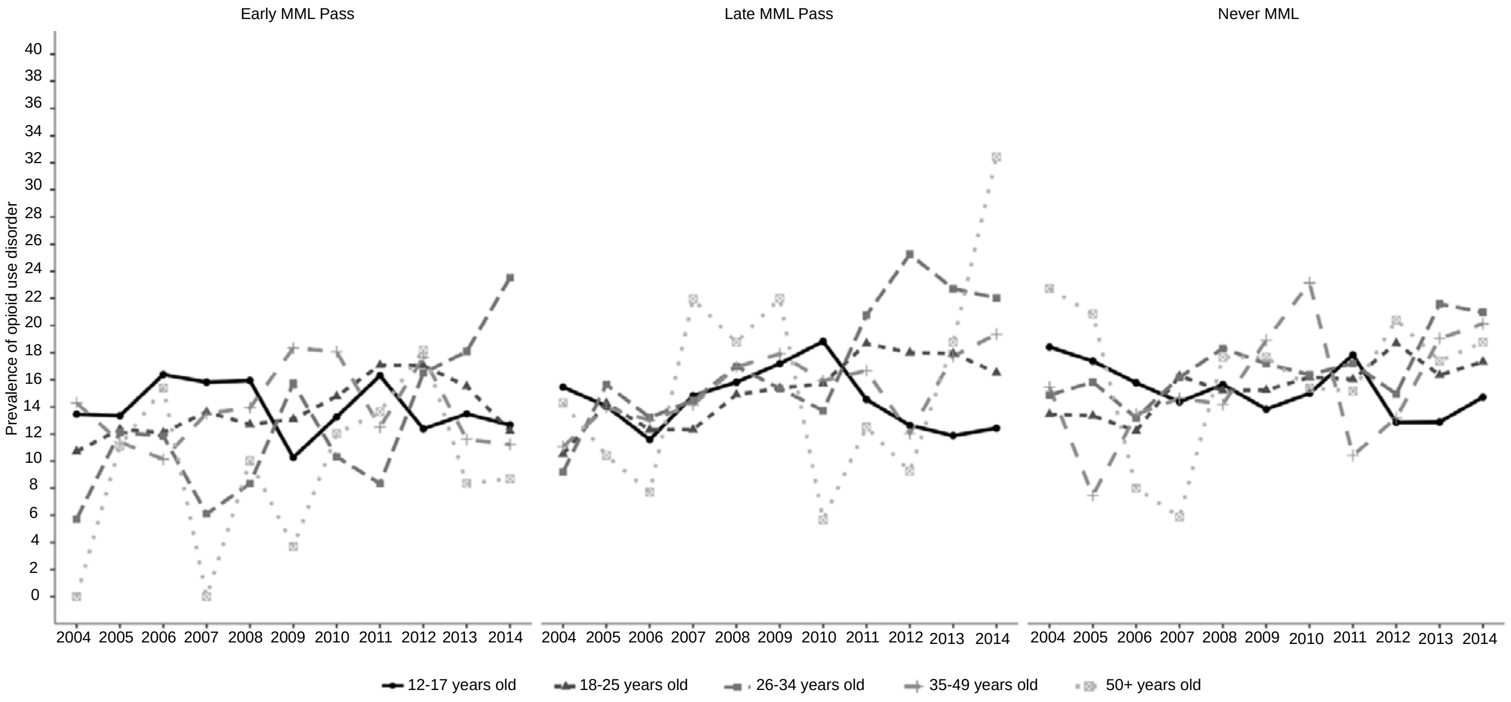

p-for-trend > 0.05 among 12-17 years old in states that never passed an MML, states that passed MML early, and states that passed MML late.

p-for-trend > 0.05 among 18-25 years old in states that never passed an MML, states that passed MML early, and states that passed MML late.

p-for-trend > 0.05 among 26-34 years old in states that never passed an MML, states that passed MML early, and states that passed MML late.

p-for-trend > 0.05 among 35-49 years old in states that never passed an MML, states that passed MML early, and states that passed MML late.

p-for-trend > 0.05 among 50+ years old in states that never passed an MML, states that passed MML early, and states that passed MML late.

MML: Medical Marijuana Laws

© 2019 Segura LE et al. *JAMA Network Open.*

eFigure 6. Prevalence of prescription opioid use disorder among prescription opioid users by race/ethnic group in states that passed MML before 2004 (Early MML Pass), after 2004 (Late MML Pass) or never passed MML.

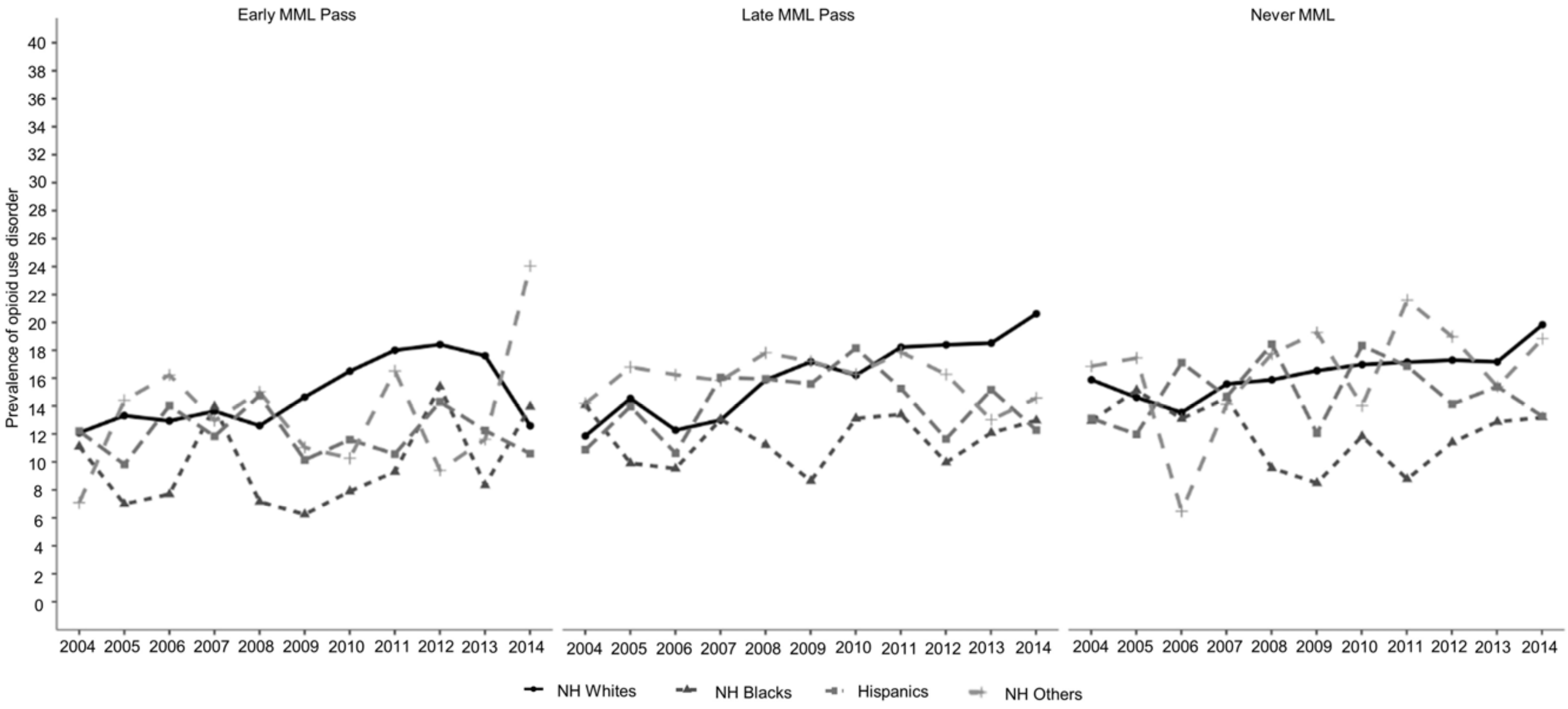

P-for-trend > 0.05 among NH Whites in states that never passed MML, states that passed MML early, and states that passed MML late.

P-for-trend > 0.05 among NH Blacks in states that never passed MML, states that passed MML early, and states that passed MML late.

P-for-trend > 0.05 among Hispanics in states that never passed MML, states that passed MML early, and states that passed MML late.

P-for-trend > 0.05 among NH Others in states that never passed MML, states that passed MML early, and states that passed MML late.

MML: Medical Marijuana Laws

© 2019 Segura LE et al. *JAMA Network Open.*

eTable 1. Comparison of past-year nonmedical prescription opioid use comparing people in states before MML passage and after MML passage without dispensaries, and between states before MML passage and after MML passage with dispensaries. NSDUH 2004-2014

	Before MML %	After MML, NO dispensaries %	Adjusted Odds Ratio (95% CI)	After MML, dispensaries %	Adjusted Odds Ratio (95% CI)
All	4.29	4.74	1.11 (1.03 - 1.20)	4.83	1.13 (1.06 - 1.22)
12 - 17 years old	5.75	5.98	1.04 (0.96 - 1.13)	6.16	1.08 (1.00 - 1.16)
18 - 25 years old	9.34	10.07	1.09 (1.01 - 1.17)	8.96	0.96 (0.89 - 1.02)
26 - 34 years old	5.99	6.18	1.03 (0.93 - 1.15)	6.31	1.06 (0.96 - 1.17)
35 - 49 years old	3.49	4.00	1.15 (1.03 - 1.29)	3.92	1.13 (1.02 - 1.25)
50+ years old	1.23	1.54	1.26 (1.04 - 1.53)	1.87	1.53 (1.30 - 1.81)
NH Whites	6.17	6.36	1.03 (0.97 - 1.11)	6.53	1.06 (1.00 - 1.14)
NH Blacks	3.47	4.36	1.27 (1.11 - 1.45)	4.33	1.26 (1.12 - 1.42)
Hispanics	3.50	3.90	1.12 (1.01 - 1.24)	4.33	1.25 (1.14 - 1.37)

Abbreviations: NSDUH: National Survey on Drug Use and Health; MML: Medical Marijuana Laws; CI: Confidence Interval; NH: Non-Hispanic.

%: prevalence.

Individual-level predictors: racial/ethnic group, age group, time as a continuous variable using a piecewise spline function of year with a knot in 2008, sex, any health insurance coverage, family income, population density based on location of individual’s household, state of residence indicator, and an indicator of living in a state with currently enacted Prescription Drug Monitoring Program laws.

State-level predictors of the proportion of the state that was White, male, ages 10-24, >25 years with at least a high school education, unemployment, and state’s median household income.

Model 1 (All) adjusted for MML status (never, before, after without dispensaries, after with dispensaries) + individual-level predictors + state-level predictors.

Model 2 (age group comparisons) adjusted for MML status (never, before, after without dispensaries, after with dispensaries) + individual-level predictors + interaction between MML status and age group + state-level predictors.

Model 3 (racial/ethnic group comparisons) adjusted for MML status (never, before, after without dispensaries, after with dispensaries) + individual-level predictors + interaction between MML status and racial/ethnic group + state-level predictors.

© 2019 Segura LE et al. *JAMA Network Open.*

eTable 2. Comparison of past-year prescription opioid use disorder among nonmedical prescription opioid users between states before MML passage and after MML passage without dispensaries, and between states before MML passage and after MML passage with dispensaries. NSDUH 2004-2014

	Before MML %	After MML, NO dispensaries %	Adjusted Odds Ratio (95% CI)	After MML, dispensaries %	Adjusted Odds Ratio (95% CI)
All	15.46	16.36	1.07 (0.89 - 1.29)	13.72	0.87 (0.72 - 1.05)
12 - 17 years old	16.09	15.19	0.93 (0.76 - 1.15)	14.84	0.91 (0.75 - 1.10)
18 - 25 years old	14.75	14.57	1.01 (0.85 - 1.21)	14.14	0.95 (0.80 - 1.13)
26 - 34 years old	17.31	15.71	0.92 (0.69 - 1.23)	13.56	0.75 (0.58 - 0.98)
35 - 49 years old	15.15	16.17	1.12 (0.82 - 1.51)	14.24	0.93 (0.70 - 1.24)
50+ years old	14.15	18.6	1.44 (0.86 - 2.41)	11.99	0.83 (0.51 - 1.33)
NH Whites	17.36	18.24	1.06 (0.90 - 1.26)	16.10	0.91 (0.78 - 1.08)
NH Blacks	12.59	11.71	0.92 (0.62 - 1.36)	10.51	0.82 (0.57 - 1.17)
Hispanics	14.74	17.62	1.24 (0.94 - 1.63)	13.37	0.89 (0.71 - 1.13)

Abbreviations: NSDUH: National Survey on Drug Use and Health; MML: Medical Marijuana Laws; CI: Confidence Interval; NH: Non-Hispanic.

%: prevalence.

Individual-level predictors: racial/ethnic group, age group, time as a continuous variable using a piecewise spline function of year with a knot in 2008, sex, any health insurance coverage, family income, population density based on location of individual's household, state of residence indicator, and an indicator of living in a state with currently enacted Prescription Drug Monitoring Program laws.

State-level predictors of the proportion of the state that was White, male, ages 10-24, ≥25 years with at least a high school education, unemployment, and state's median household income.

Model 1 (All) adjusted for MML status (never, before, after without dispensaries, after with dispensaries) + individual-level predictors + state-level predictors.

© 2019 Segura LE et al. *JAMA Network Open.*

Model 2 (age group comparisons) adjusted for MML status (never, before, after without dispensaries, after with dispensaries) + individual-level predictors + interaction between MML status and age group + state-level predictors.

Model 3 (racial/ethnic group comparisons) adjusted for MML status (never, before, after without dispensaries, after with dispensaries) + individual-level predictors + interaction between MML status and racial/ethnic group + state-level predictors.

© 2019 Segura LE et al. *JAMA Network Open.*

eTable 3. Sensitivity Analyses to quantify the minimum amount of unmeasured confounding required to nullify the associations between NMUPO and MML passage, and POUD among PO users and MML passage.

E-value: the minimum strength of association that an unmeasured confounder would need to have with both the exposure (MML) and the outcome (NMUPO), above and beyond the measured confounders, to make the exposure-outcome association (aOR)—E-value for the aOR—and the CI lower limit (E-value for the CI lower limit) null.

NMUPO before versus after MML passage			
	aOR after vs before (95% CI)	E-value for the aOR	E-value for the CI Lower Limit
All	1.13 (1.06, 1.20)	1.51	1.31
35-49 years old	1.15 (1.05, 1.25)	1.57	1.28
50+ years old	1.42 (1.23, 1.64)	2.19	1.76
NH Blacks	1.26 (1.14, 1.39)	1.83	1.54
Hispanics	1.21 (1.11, 1.31)	1.71	1.46
NMUPO before versus after MML passage without dispensaries			
All	1.11 (1.03, 1.20)	1.46	1.21
18-25 years old	1.09 (1.01, 1.17)	1.4	1.11
35-49 years old	1.15 (1.03, 1.29)	1.57	1.21
50+ years old	1.26 (1.04, 1.53)	1.83	1.24
NH Blacks	1.27 (1.11, 1.45)	1.86	1.46
Hispanics	1.12 (1.01, 1.24)	1.49	1.11
NMUPO before versus after MML passage with dispensaries			
All	1.13 (1.06, 1.22)	1.51	1.31
35-49 years old	1.13 (1.02 - 1.25)	1.51	1.16
50+ years old	1.53 (1.30 - 1.81)	2.43	1.92
NH Blacks	1.27 (1.12 - 1.42)	1.86	1.49
Hispanics	1.25 (1.14 - 1.37)	1.81	1.54
POUD among PO users before versus after MML passage with dispensaries			
26-34 years old	0.75 (0.58 - 0.98)	2	1.17

© 2019 Segura LE et al. *JAMA Network Open.*

NMUPO: Nonmedical use of prescription opioids. MML: Medical Marijuana Laws. POUD: Prescription opioid use disorder.			
PO: Prescription opioid. NH: Non-Hispanic. CI: Confidence Interval. aOR: adjusted Odds Ratio.			

© 2019 Segura LE et al. *JAMA Network Open.*
